# Liver imaging features by convolutional neural network to predict the metachronous liver metastasis in stage I-III colorectal cancer patients based on preoperative abdominal CT scan

**DOI:** 10.1186/s12859-020-03686-0

**Published:** 2020-09-17

**Authors:** Sangwoo Lee, Eun Kyung Choe, So Yeon Kim, Hua Sun Kim, Kyu Joo Park, Dokyoon Kim

**Affiliations:** 1 Division of Future Convergent, The Cyber University of Korea, Seoul, 03051 South Korea; 2 Department of Surgery, Seoul National University Hospital Healthcare System Gangnam Center, Seoul, 06236 South Korea; 3 Department of Biostatistics, Epidemiology & Informatics, Perelman School of Medicine, University of Pennsylvania, B304 Richards Building, 3700 Hamilton Walk, Philadelphia, PA 19104-6116 USA; 4 Department of Software and Computer Engineering, Ajou University, Suwon, 16499 South Korea; 5 Department of Radiology, Seoul National University College of Medicine, Seoul, 03080 South Korea; 6 Department of Surgery, Seoul National University College of Medicine, Seoul, 03080 South Korea; 7 Institute for Biomedical Informatics, University of Pennsylvania, Philadelphia, PA 19104 USA

**Keywords:** Radiomics, Colorectal cancer, Convolutional neural network, Artificial intelligence

## Abstract

**Background:**

Introducing deep learning approach to medical images has rendered a large amount of un-decoded information into usage in clinical research. But mostly, it has been focusing on the performance of the prediction modeling for disease-related entity, but not on the clinical implication of the feature itself. Here we analyzed liver imaging features of abdominal CT images collected from 2019 patients with stage I – III colorectal cancer (CRC) using convolutional neural network (CNN) to elucidate its clinical implication in oncological perspectives.

**Results:**

CNN generated imaging features from the liver parenchyma. Dimension reduction was done for the features by principal component analysis. We designed multiple prediction models for 5-year metachronous liver metastasis (5YLM) using combinations of clinical variables (age, sex, T stage, N stage) and top principal components (PCs), with logistic regression classification. The model using “1^st^ PC (PC1) + clinical information” had the highest performance (mean AUC = 0.747) to predict 5YLM, compared to the model with clinical features alone (mean AUC = 0.709). The PC1 was independently associated with 5YLM in multivariate analysis (beta = − 3.831, *P* < 0.001). For the 5-year mortality rate, PC1 did not contribute to an improvement to the model with clinical features alone. For the PC1, Kaplan-Meier plots showed a significant difference between PC1 low vs. high group. The 5YLM-free survival of low PC1 was 89.6% and the high PC1 was 95.9%. In addition, PC1 had a significant correlation with sex, body mass index, alcohol consumption, and fatty liver status.

**Conclusion:**

The imaging features combined with clinical information improved the performance compared to the standardized prediction model using only clinical information. The liver imaging features generated by CNN may have the potential to predict liver metastasis. These results suggest that even though there were no liver metastasis during the primary colectomy, the features of liver imaging can impose characteristics that could be predictive for metachronous liver metastasis.

## Background

In colorectal cancer (CRC) patients, liver is the most common site of metastasis due to its anatomical connection with portal circulation [1]. In advanced CRC, liver may be the sole site of metastasis in 30–40% of patients [2, 3]. In these patients, median survival is 5–20 months without intervention, and 5-year survival is extremely rare [4]. Thus, understanding the pathophysiology of the liver metastasis is one of the most critical subjects for CRC management.

Radiomics is an innovative technique that uses the large volume of imaging features to predict oncological features [5]. It consists of converting the medical images into high-dimensional features which is then used to predict clinical outcomes [6].Convolutional neural networks (CNNs) have recently manifested the ability to generate useful features from imaging data in various medical research [7]. There are multiple studies suggesting the CT imaging features extracted by CNNs have high predictive values in oncological outcomes [8, 9]. Machine learning approach is one of the major subfields of artificial intelligence which can be used for constructing prediction model in radiomics [6] and has shown promising performances for predicting various oncological subjects [10–13].

Nonetheless, there are challenges that to evaluate the values of each imaging feature in the prediction model for an oncological outcome is difficult [14]. In traditional approach for radiological research, to review the medical images, radiologists use the human visual interpretation based on the characteristics of the images systematized by accumulated experiences and researches in association with clinical manifestations [15] and the statistical models are used to find associations in these data that could enhance clinical reasoning [16]. This canonical way to use medical images for clinical research might be presenting challenges to radiomics, which uses the imaging features generated by CNNs and designs prediction models by machine learning to enhance clinical performances. If the approaches of radiomics study can encompass purposes focusing on not only the performance of the prediction modeling for disease-related entity, but also the clinical implication of the imaging features itself, its utility and contribution to the healthcare research would be in great value.

Here we propose a framework to extract the liver imaging features from CT scan using CNNs in CRC patients and analyze the imaging features using machine learning approaches to predict the metachronous liver metastasis. Additionally, we tried to elucidate its clinical implication in oncological perspectives using statistical analysis. As a proof of concept study, abdominal CT images were collected from 2019 patients who had colectomy for stage I–III CRC, preoperatively.

## Methods

### Patients and data acquisition

We performed a retrospective, cross-sectional study in patients who underwent colectomy for CRC from January 2008 to September 2013 at Seoul National University Hospital. Stage I-III CRC patients who had curative resection were included in the analyses. The exclusion criteria consisted of patients who had surgery less than 5 years; recurrence in or distant metastasis to other than liver after in less than 5 years after surgery; preoperative neoadjuvant chemotherapy; a history of liver resection; have liver lesion; and had a poor quality of preoperative CT scan. A total of 2019 patients were eligible, and their electronic medical records (EMR) and CT images were collected. The CRC staging description was based on the AJCC staging system, seventh edition, which is a classification system provided by the American Joint Committee on Cancer for describing the extent of cancer progression [17]. We used the clinical information turned into a dichotomized form such as T stages into T1, T2 vs. T3, T4, N stages into N0 vs. N1, N2 and patient ages into < 65 years vs. > = 65 years. 5-year metachronous liver metastasis (5YLM) rate was the primary endpoint. If the patient died or had recurrence other than in liver, they would not be counted as metachronous liver metastasis incidences. We used the abdominal CTs taken before the colectomy for clinical staging. For image acquisition, the non-contrast abdominal CT scan image of each patient was used. A physician (author EKC) acquired the images under the guidance of radiologist (author HSK). Cross-sectional images at the level where the caudate lob of the liver is most prominent were selected with abdominal view setting. The regions of interest (ROIs) were placed at the segment 7 of the Couinaud system [18], which is the right – posterior – superior segment of liver, with a size of 50 × 50 pixels.

### Feature generation

**Fig. 1:**
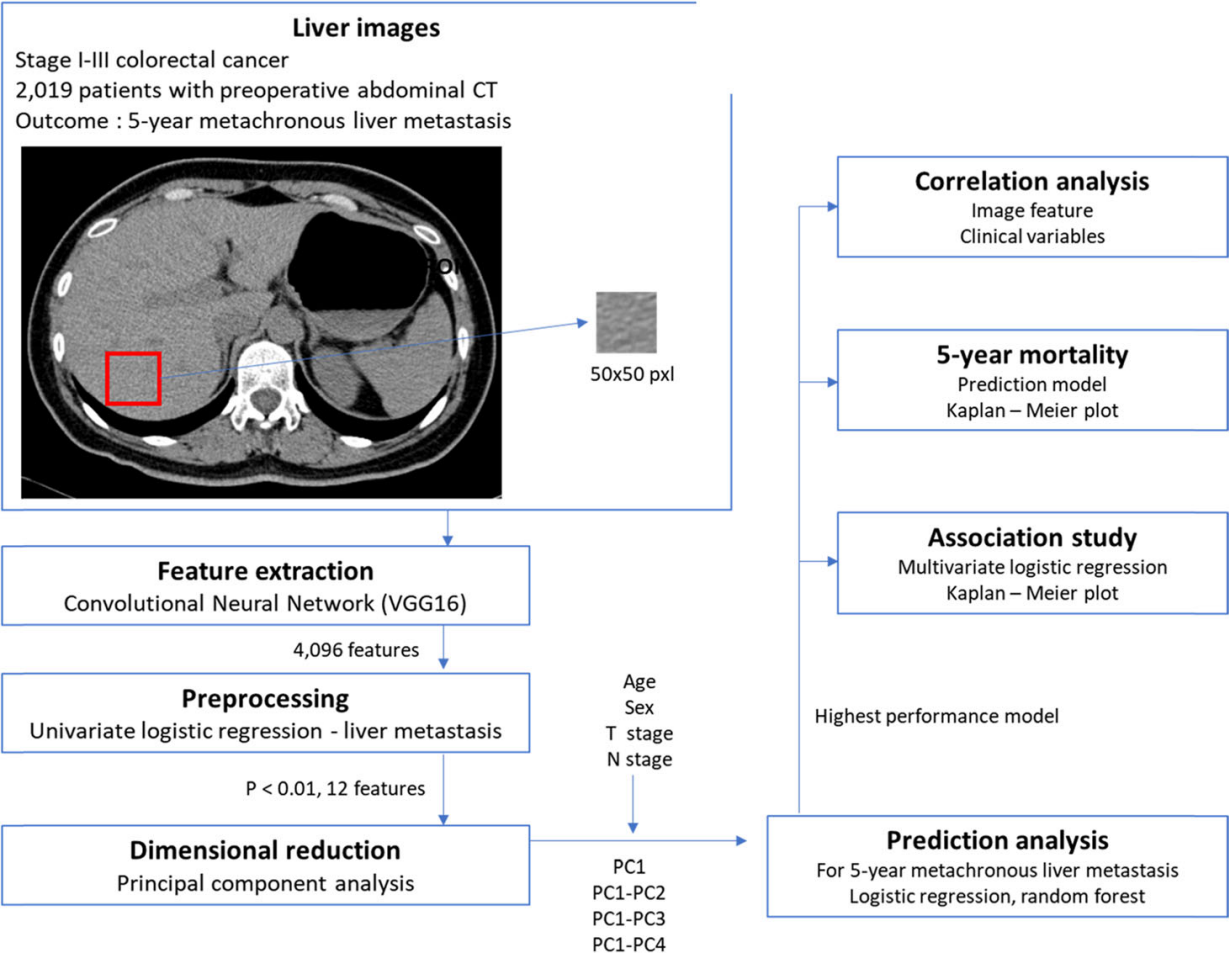
Study design. Overview of the analysis framework. Feature extraction on the abdominal CT, 50 × 50 pixel ROIs, was done by utilizing a pre-trained convolutional neural network. We preprocessed them based on the significance of association with 5-year liver metastasis (5YLM) rate by performing univariate logistic regression analysis. Principal component analysis (PCA) was done for feature reduction in dimensionality and this generated new sets of feature. We used two machine learning algorithms, such as logistic regression classification (LR) and random forest classification (RFC) to train prediction models for 5YLM and compared the performances of each model. Among the models to predict 5YLM, we used the highest AUC model to perform multivariate logistic regression to association between the image features and 5YLM statistically. Then Kaplan Meier analysis was done by the principal components (PCs) for metachronous liver metastasis free survival and overall survival. We done a correlation analysis between the significant PCs and the clinical variable in Table 1. We also applied the highest AUC model for 5YLM to predict 5-year mortality and observed whether the liver image feature could do a predictive role for 5-year mortality

Figure 1 shows the overview of the analysis framework. First, feature extraction on the images was done by utilizing a pre-trained convolutional neural network, VGG16 [19], which do not require further training. For the feature extraction, we used the fully connected layers, which is a top-layer of the pre-trained model of VGGnet with 16 layers (VGG-16). The 4096 features were extracted as an output. Then, we preprocessed the extracted imaging features rather than using whole 4096 features based on the significance of association with 5YLM rate by performing univariate logistic regression analysis. The subsets of imaging features that passed the suggestive significance level (*P* < 0.01) were used for further analyses. Lastly, principal component analysis (PCA) was performed for reducing the feature dimensionality, and this generated new sets of features, such as 1st principal component (PC1) to 10th principal component (PC10), sequentially. At the input stage to the machine learning methods, we further standardized either the clinical features or the PC-transformed image features, by z normalization for each feature to have a mean of zero and unit variance.

### Prediction models

We trained two machine learning algorithms, including logistic regression classification (LR) and random forest classification (RFC) to predict 5YLM and compared the performances of each model. The models were designed by respective combination of features consisting of clinical features, which includes age, gender, T stage and N stage, and imaging features, which are the sequential summation of PC1 to PC10. The whole data set was divided into a training set (70%) and a test set (30%), and a five-fold cross validation scheme was used on the training set for the parameter tuning of the classification models. Given our highly imbalanced data set, we adopted five-fold, considering that five can be reasonable to allow enough number of positive data samples in each fold. Due to the highly imbalanced data set, we dealt with the imbalance problem of the training set first by oversampling on the negative minority class to meet negative/positive ratio 0.2, followed by down-sampling on the positive majority class to meet negative/positive ratio 0.4 finally. For up-sampling, we used SMOTE (Synthetic Minority Over-Sampling Technique) [20], and for down-sampling, we used the random down-sampling technique. After performing five-fold cross-validation on the training set, the trained model is validated on the test set. We note that cross-validation procedure is used to prevent overfitting to the training set when fitting the prediction model (LR and RFC). To validate the trained model, we performed the procedure aforementioned (splitting the data set into a training set and a test set; SMOTE followed by down-sampling; a five-fold cross validation to find the optimal parameters; and performance evaluation on the test set) for 100 iterations. Using the trained model, we evaluated the performance of the test set. The performances were measured by the area under the curve (AUC) of the receiver operating characteristics (ROC) curve and presented as means and standard deviations.

We also applied the model with the highest AUC for 5YLM to predict 5-year mortality and observed whether the liver imaging feature contribute to a prediction of 5-year mortality. The AUC performance was compared with the model using clinical feature only for the prediction of 5-year mortality.

### Association study

Among the models to predict 5YLM, we used the model with the highest AUC to perform multivariate logistic regression to identify associations between the imaging features and 5YLM. Then, Kaplan-Meier analysis was conducted based on the principal components (PCs) that were significantly associated with metachronous liver metastasis-free survival and overall survival (*P* < 0.05). For survival analysis, the patients excluded by the previous exclusion criteria, such as recurrence in or distant metastasis to other than liver after in less than 5 years after surgery, were included. The optimal cut-off points for those PCs to divide patients into two groups were determined by MaxStat packages in R (Maximally selected Rank Statistics). MaxStat uses the maximally selected rank statistics to recommend the optimal cut-off point for the survival plot [21]. Univariate cox proportional hazard regression analysis was performed to compare the differences between PC-based groups using the time to event and the censoring data of it.

### Correlation study

To investigate the clinical relevance of the imaging features, we ran a correlation analysis between the significant PCs and the additional clinical variables in Table 1, such as age, sex, body mass index, tumor location, alcohol consumption, liver function test, fatty liver status, T stage, N stage, Angiolymphatic invasion venous invasion, and postoperative follow-up duration. We measured Pearson’s correlation between numeric variables, and Spearman’s correlation between categorical variables, respectively. *P* value of the correlation coefficient between variables less than 0.05 was considered statistically significant. The results were visualized with “corrplot” R package.

**Table 1:** Demographic features of the study population

	Liver metastasis, no (***N*** = 1919, 95.04%)	Liver Metastasis, yes (***N*** = 100, 4.96%)	*P* value
Age (years)	62.3 ± 9.2	63.1 ± 9.5	0.390
Age > =65 years			0.640
No	1092 (56.9%)	54 (54.0%)	
Yes	827 (43.1%)	46 (46.0%)	
Sex			0.727
Male	1204 (62.7%)	65 (65.0%)	
Female	715 (37.3%)	35 (35.0%)	
BMI (kg/m2)	23.9 ± 3.0	23.8 ± 3.1	0.805
BMI (> = 25 kg/m2)			0.648
No	1280 (66.7%)	64 (64.0%)	
Yes	638 (33.3%)	36 (36.0%)	
Tumor location			0.127
Right	501 (26.4%)	19 (19.0%)	
Left	1397 (73.6%)	81 (81.0%)	
Heavy alcohol consumption			1
No	1261 (65.7%)	66 (66.0%)	
Yes	658 (34.3%)	34 (34.0%)	
GOT	22.3 ± 9.0	21.8 ± 9.6	0.559
GPT	20.3 ± 12.8	19.8 ± 16.0	0.737
Fatty liver			0.283
No	1743 (95.0%)	96 (98.0%)	
Yes	91 (5.0%)	2 (2.0%)	
T stage			< 0.001
T1 stage	359 (18.7%)	4 (4.0%)	
T2 stage	352 (18.3%)	6 (6.0%)	
T3 stage	1106 (57.6%)	74 (74.0%)	
T4 stage	102 (5.3%)	16 (16.0%)	
N stage			< 0.001
N0	1293 (67.4%)	28 (28.0%)	
N1	463 (24.1%)	39 (39.0%)	
N2	163 (8.5%)	33 (33.0%)	
Lymph node metastasis			< 0.001
Absent	1293 (67.4%)	28 (28.0%)	
Present	626 (32.6%)	72 (72.0%)	
Overall stage			< 0.001
Stage 1	581 (30.3%)	6 (6.0%)	
Stage 2	658 (34.3%)	18 (18.0%)	
Stage 3	680 (35.4%)	76 (76.0%)	
Angiolymphatic invasion			< 0.001
Absent	1434 (77.0%)	57 (57.6%)	
Present	429 (23.0%)	42 (42.4%)	
Venous invasion			< 0.001
Absent	1729 (92.8%)	75 (75.8%)	
Present	134 (7.2%)	24 (24.2%)	
Postoperative follow up duration	1893.5 ± 767.7	1554.5 ± 784.9	< 0.001
5-year Mortality			
Alive	1919 (100.0%)	61 (61.0%)	< 0.001
Dead	0 (0.0%)	39 (39.0%)	

All the statistical and computational analyses were done by R statistical software (version 3.5.3 R) and Python software (version 3.6.2). Associations between clinical information and 5YLM rate were assessed by Chi-square test, Student’s t-test, and analysis of variance (ANOVA) for independent groups in Table 1.

### Ethics statement

The Institutional Review Board of Seoul National University Hospital approved the study protocol (IRB number 1902–088-1010), and the study was conducted in accordance with the Declaration of Helsinki. Informed consent was waived by the board.

## Results

### Patient demographics

Our study sample comprised 2019 patients (1269 males and 750 females) who had colectomy for stage I-III colorectal cancer. The mean patient age was 62.32 +/− 9.21 years. There were 100 cases (4.96%) of metachronous liver metastasis during the follow. Patient characteristics are shown in Table 1.

### 5-year metachronous liver metastasis prediction based on principal components and clinical information

We preprocessed the extracted imaging features from CNN based on the significance of association with 5YLM rate by performing univariate logistic regression analysis. Twelve features passed the suggestive significance level (*P* < 0.01). From the 12 features, using the PCA, we generated new sets of features notated as PC1 to PC10.

For 5YLM prediction models, we used PCs and clinical features as inputs to our models, in which we not only incremented the number of PCs one by one (for example, PC1, PC1-PC2, PC1-PC3 and so on), but also adopted various combinations (for example, clinical feature only model, PCs only model, clinical feature plus PC model) in order to validate which combination of features more contribute to the prediction performance. Two supervised machine learning methods, LR and RFC, were used for the performance evaluations.

The performances of each model are shown in Table 2. Model with 1st PC (PC1) showed the highest performance among other PCs combinations both in LR (mean AUC = 0.606) and RFC (AUC = 0.557). In the combination of clinical and imaging features, LR model trained with PC1 and clinical features showed the best performances (mean AUC = 0.747), which imply that the model using the imaging features in combination with the clinical features improved the prediction performance rather than the model using clinical feature only (mean AUC = 0.709).

**Table 2:** Performances of the prediction models in the test set for 5-year mortality and 5-year metachronous liver metastasis

Predictors	Logistic regression classification AUC (mean +/− standard deviation)	Random forest classification AUC (mean, standard deviation)
Prediction model for 5-year metachronous liver metastasis		
Clinical*	0.709 +/− 0.038	0.692 +/− 0.038
PC1	0.606 +/− 0.044	0.557 +/− 0.043
PC1-PC2	0.600 +/− 0.042	0.536 +/− 0.042
PC1-PC3	0.588 +/− 0.040	0.503 +/− 0.046
PC1-PC4	0.580 +/− 0.040	0.520 +/− 0.042
**Clinical + PC1**	**0.747 +/− 0.036**	0.697 +/− 0.038
Clinical + PC1-PC2	0.744 +/− 0.036	0.676 +/− 0.043
Clinical + PC1-PC3	0.740 +/− 0.038	0.668 +/− 0.042
Clinical + PC1-PC4	0.736 +/− 0.038	0.691 +/− 0.042
Prediction model for 5-year mortality		
Clinical*	0.704 +/− 0.028	0.679 +/− 0.030
PC1	0.482 +/− 0.031	0.511 +/− 0.030
Clinical + PC1	0.695 +/− 0.031	0.647 +/− 0.033

*Clinical: Age, Sex, T stage, N stage

### Association study with the oncological and clinical variables using the designed model

For the model with PC1 and clinical features (age, sex, T stage, and N stage), which is the best performed model, an association study was done to investigate the association between the features and 5YLM. By multivariate logistic regression analysis, PC1 showed independent association with 5YLM, significantly (beta = − 3.831, *P* < 0.001) (Table 3).

**Table 3:** Multivariate logistic regression analysis for 5-year metachronous liver metastasis

	Beta (standard error)	*P* value
**Using PCs from 12 features**		
Age (> = 65 years)	0.119 (0.213)	0.213
Gender (Female)	−0.232 (0.223)	0.297
T3, T4 stage	1.276 (0.345)	< 0.001
N1, N2 stage	1.467 (0.234)	< 0.001
**PC1**	**−3.831 (1.012)**	**0.0001**

**Fig. 2:**
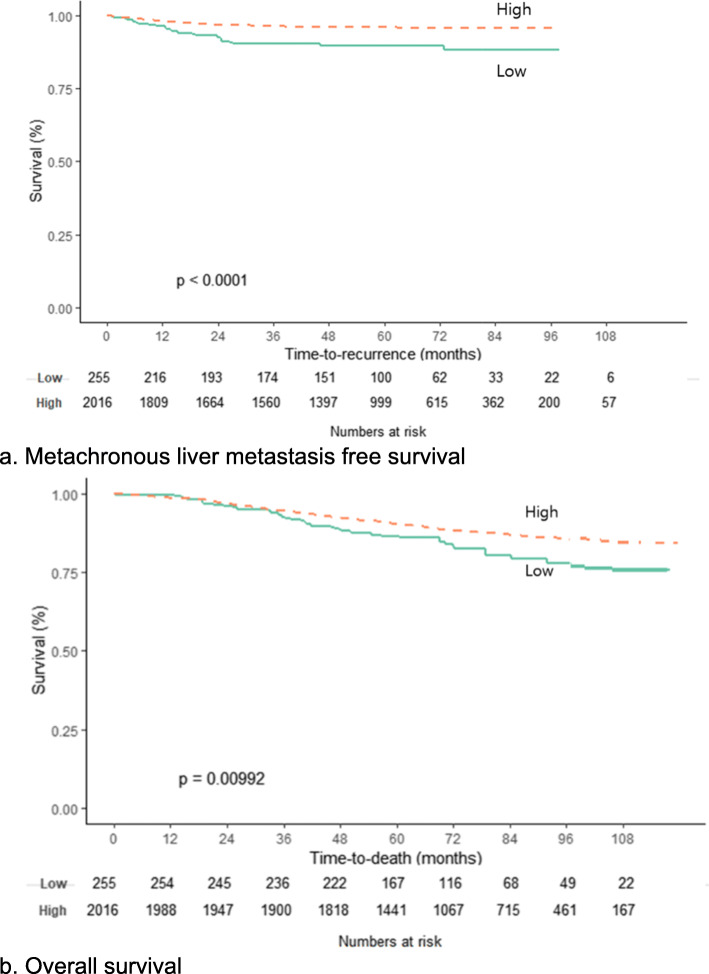
Kaplan Meier plots for metachronous liver metastasis free survival and overall survival using 1st PC of image features. **a**. Metachronous liver metastasis free survival The populations were divided by the optimal cut offs for PC1 score based on MaxStat (− 0.135). The difference between two group was compared by univariate cox proportional hazard regression. The 5-year metachronous liver metastasis free survival of low group (PC1 score below − 0.135) was 89.6% and the high group (PC 1score above − 0.135) was 95.9%. **b**. Overall survival Using the same PC1 group, K-M plot was visualized for overall survival

For the PC1, Kaplan-Meier plots were generated for metachronous liver metastasis free survival (Fig. 2a). The patients were divided by the optimal cut offs for each PC1 based on MaxStat. (− 0.135 for the PC1 score). The results are shown in Fig. 2 with the result of univariate cox proportional hazard regression. The 5YLM-free survival of low group (PC1 score below − 0.135) was 88.7% and the high group (PC 1score above − 0.135) was 95.6%. We also evaluated with the Kaplan-Meier plot for overall survival and the results were similar that low group had poor overall survival compared to high group, significantly (*P* < 0.001) (Fig. 2b).

**Fig. 3:**
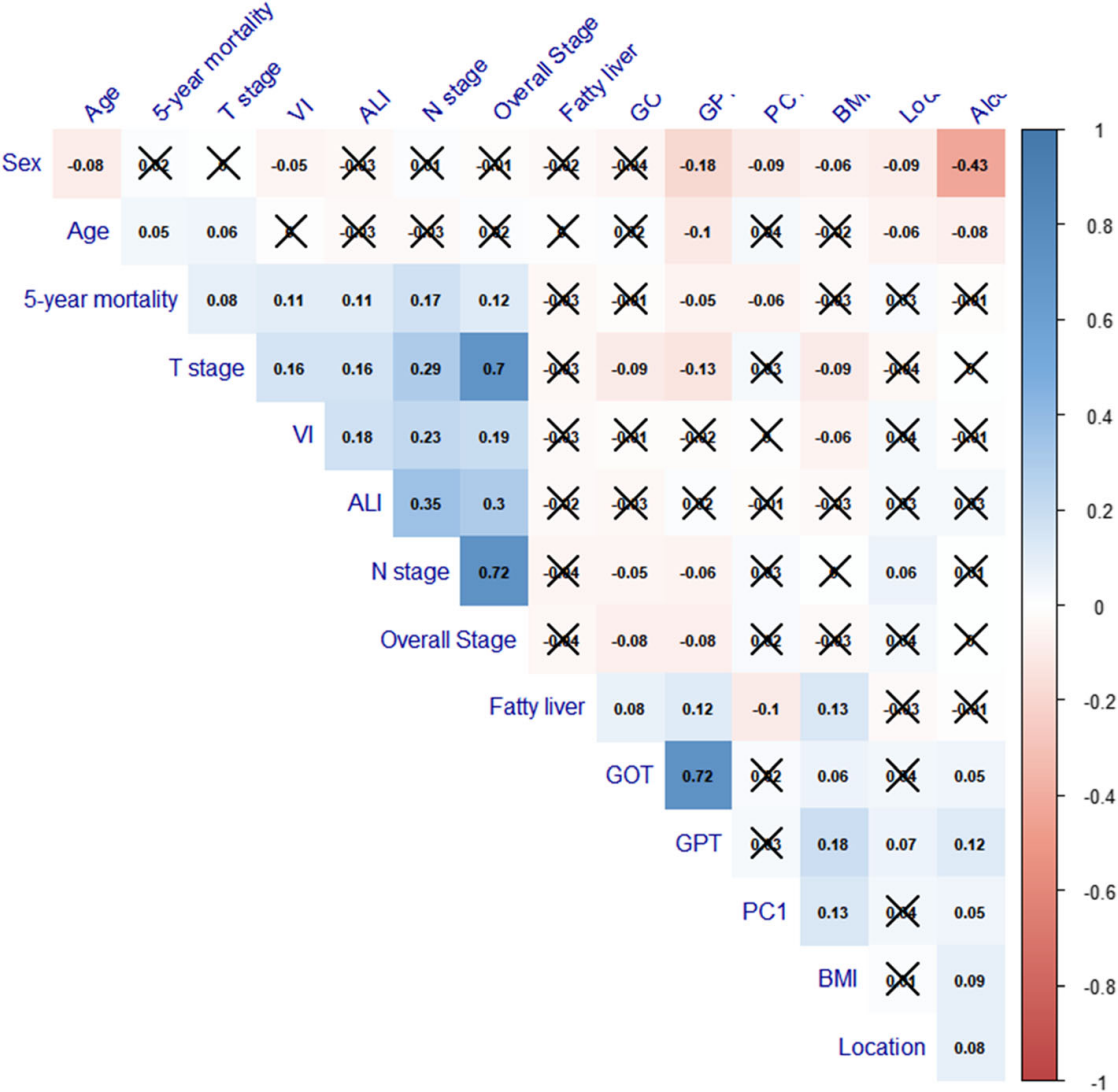
Correlation plots for 1st PC and clinical variables. Correlations with *p*-value > 0.05 are considered as insignificant. In this case the correlation coefficient values are leaved blank or crosses are added. 1st PCA had significant correlation with sex, body mass index, alcohol consumption and fatty liver status

In the correlation analysis between the significant PC1 and the clinical variable in Table 1, sex, body mass index, alcohol consumption and fatty liver status had significant correlation with PC1 (Fig. 3).

### Performance of the prediction model in 5-year mortality prediction

We applied the 5YLM prediction model with the highest AUC model, which combined clinical features with PC1, with respect to the prediction of 5-year mortality. In the 5-year mortality prediction model, the liver imaging features did not have an additional predictive power (mean AUC =0.690), when compared with clinical only models (mean AUC = 0.700), in both models, trained with LR and RFC.

## Discussion

Currently, clinical features, such as age, sex, T stage, and N stage are most commonly used to predict the prognosis of colorectal cancer survival [22]. Comparing the prediction performance of these clinical features, the integrative model that integrates clinical information and imaging features by convolutional neural network significantly improved performance of prediction for 5YLM rate.

This is quite interesting result because adding the 50 × 50 pixel liver imaging features would contain extremely limited information, but still, we demonstrated the additional imaging features when combined with clinical features improved prediction performance of liver metastasis. The underlying mechanism would be that, though at the time of surgery the liver does not seem to impose liver metastasis on gross finding of CT images, there could be underlying molecular changes in liver that could be predictive of liver metastasis. For 5-year mortality, the liver feature did not have additive predictive power in prediction, but there was a significant association with overall survival in K-M plot. In our study, we applied both statistical association analysis and computational predictive analysis for the outcome. Association studies would focus on understanding a relationship between the variables and outcomes, while prediction studies train the model with the training data to obtain the variables to investigate their predictive power for the corresponding outcome. Association studies might provide explanation for the relationship but might not have predictive power. On the contrary, prediction studies might have high performance but hard to interpret it. This might imply that the liver image features alone are not enough to precisely predict the outcome of 5-year mortality, however they are associated with the 5-year mortality based on the results of Kaplan-Meier survival analysis with significant *p*-values.

Metachronous liver metastasis significantly influence the prognosis of CRC patients who had curative colectomy [16, 23], and it is reported that in 20–30% of patients, it will be detected following primary colectomy [24–26]. Well-known risk factors for liver metastasis are N stage, vascular invasion and preoperative carcinoembryonic antigen (CEA) level [23, 27, 28]. Additionally, there are also suggested factors predisposing to the development of metachronous liver metastasis in CRC, such as tissue micro-environmental changes and chronic inflammation [29]. In the present study, we focused on investigating the clinical relevance of this imaging features performing association and correlation studies with patient’s demographical and oncological information. The representative imaging feature, the 1st principal component (PC1), had a significant association with 5YLM-free survival, and the results were shown in Kaplan-Meier plot. In the correlation study, PC1 had significant correlations with sex, body mass index, alcohol consumption and fatty liver status. Sex [30], body mass index [29], alcohol consumption [31] and fatty liver status [32] are reported to be correlated with liver metastasis in CRC. Obesity determined by body mass index change or sex difference and hepatosteatosis derivative of fatty liver or alcoholism are suggestive predisposing factors in the function of tissue microenvironment change and chronic inflammation [29]. It can be postulated that the imaging features of liver ROIs impose various heterogeneity of predisposing factors for metachronous liver metastasis comprehensively.

In the study design, we additionally preprocessed the 4096 imaging features extracted by training the convolutional neural network, VGG16, based on the significance of association with 5YLM rate. VGG16 is a convolutional neural network model which achieved 92,7% accuracy in ImageNet, a dataset of over 14 million images belonging to 1000 classes, and it used RGB images for training [19]. This model can capture complex features like human faces, natural scenes and showed human level performance [33]. The preprocessing step was introduced because designing the prediction model with the whole 4096 imaging features from CNN did not provide good results for 5YLM (data not shown). This might come from the fact that pretrained CNN basically trained RGB images which has chromatic color, shape and size variance but in CT scan image, it is achromatic color, fixed square shape and fixed 50 × 50 pixel sized. Thus, preprocessing with statistical association threshold will remove the noises and include only the effective features to be introduced.

With the preprocess features, principal component analysis was performed for reducing the feature dimensionality, and new sets of features, such as 1st principal component (PC1) to 10th principal component (PC10) as an imaging features for the prediction model and showed that PC1 had improved the performance of prediction model by clinical information to predict 5YLM. We used the PC transformed as imaging features rather than the ones after preprocessing, which is primarily generated by CNN, because these did not have promising results in the prediction model (data not shown). This might reflect the fact that the respective imaging feature by itself do not have meaningful contribution for prediction but the selected features should be aggregated to have a predictive role in the model.

In our study, we used logistic regression and random forest classification, which are very common and widely studied machine learning models [34]. For the prediction of 5YLM, the model trained by logistic regression showed the improvement by adding imaging features to the clinical features compared with the ones only consisting of clinical features. However, the improvement of performances was modest by the models trained by random forest classification. Logistic regression is a classical machine learning classifier and it has advantage of having fast speed to train the inputs and making it more interpretable [6, 35]. In a recent study, comparing logistic regression and random forest classification for binary outcomes, when increasing the variance in the explanatory and noise variables, logistic regression consistently better performed as compared to random forest classification [34]. However, true positive rate was higher in random forest classification compared to logistic regression. When machine learning is applied to the clinical fields, the characteristics of the data set and the purpose of using machine learning should be considered carefully before choosing which algorithms to be applied.

This study has several advantages. First, we comprehensively analyzed the imaging feature by respective combination of image features and clinical information. By this way, we were able to find that the best-performing combination of imaging features, preprocessed features by association analysis and PCs from PCA analysis, which showed improved prediction performance combined with clinical information. Second, we performed both the prediction model based on computational analysis and association and correlation study based on statistical analysis. This will help to interpret the complex nature of the liver CT scan image features more intuitively, provide the clinical relevance of it and support an evidence for the results of prediction model. Third, all the patients enrolled in the analyses had at least 5 years of follow-up after primary colectomy. This will make the results of analyses more reliable since the information involves the long-term observations.

Despite demonstrating the validity of our proposed approach, there might be a couple of remaining potential limitations. First, as we used the retrospectively collected CT scan, we could not collect the parameters and the product information of the CT scanner, which could influence the imaging features as a batch effect. But since, the CT images were taken on the purpose of preoperative clinical staging in colorectal cancer, the quality of the CT images was well controlled. Second, we simply concatenated the clinical factors and imaging features level for integration. Transformational integration could be applied in a larger set of samples [36]. Third, since there are no open source databases that includes both abdominal CT images and clinical information, we could not replicate the results in another data set.

## Conclusion

By analyzing the liver image features by convolutional neural network in stage I-III colorectal cancer patients based on preoperative abdominal CT scan, we were able identify the contribution of imaging features to predict the metachronous liver metastasis. The 1st PC from imaging feature combined with clinical information improved the performance of standardized prediction model using only clinical information such as age and TNM stage. The preoperative liver imaging features generated by CNN might have the potential to predict liver metastasis after 5 years. This result suggests that even though there were no metastasis or liver lesion during the primary colectomy for stage I-III CRC, the features of liver in preoperative CT scan can impose characteristics that could be predictive for liver metastasis during the postoperative follow-up. The findings might conceptualize the importance of the imaging features in liver which could be applied for clinical practice.

## Data Availability

The datasets generated and/or analyzed during the current study are not publicly available due to restrictions (the institution policy to protect the privacy of research participants) but are available from the corresponding author on reasonable request.

## Abbreviations

CRC: Colorectal cancer
CNN: Convolutional neural network
5YLM: 5-year metachronous liver metastasis
PCs: Principal components
AUC: Area under the curved
VGG-16: VGGnet with 16 layers
PCA: Principal component analysis
PC1: 1st principal component
PC10: 10th principal component
LR: Logistic regression
RFC: Random forest classification
SMOTE: Synthetic Minority Over-Sampling Technique
